# Risk of AL Amyloidosis is Associated with Degree of Free Light Chain Elevation and Duration of Exposure

**DOI:** 10.21203/rs.3.rs-9227260/v1

**Published:** 2026-04-08

**Authors:** Angela Dispenzieri, Maximilian Steinhardt, Eli Muchtar, Taxiarchis Kourelis, Rahma Warsame, Francis Buadi, David Dingli, Nelson Leung, Joselle Cook, Ronald Go, Suzanne HAYMAN, Wilson Gonsalves, Prashant Kapoor, Saurabh Zanwar, Moritz Binder, Tamer Hellou, Amie Fonder, Miriam Hobbs, Nadine Abdallah, Yi Lin, Mustaqeem Siddiqui, Robert KYLE, Martin Kortüm, Hermann Einsele, S. Vincent Rajkumar, Shaji Kumar, Morie Gertz

**Affiliations:** Mayo Clinic; Mayo Clinic; Mayo Clinic; Mayo Clinic; Mayo Clinic; Mayo Clinic; Mayo Clinic; Mayo Clinic; Mayo Clinic; Mayo Clinic; Mayo Clinic; Mayo Clinic; Mayo Clinic; Mayo Clinic; Mayo Clinic; Mayo Clinic; Mayo Clinic; Mayo Clinic; Mayo clinic; Mayo Clinic; Mayo Clinic; Mayo Clinic; University Hospital of Würzburg; University Hospital Würzburg; Mayo Clinic; Mayo clinic; Mayo Clinic

## Abstract

Systemic light chain amyloidosis (AL) arises from monoclonal immunoglobulin light chains, but determinants of progression from precursor states remain poorly defined.

In a cross-sectional cohort comprising 1950 systemic AL patients diagnosed 2010-2024, 258 (13.2%) patients with a previously diagnosed plasma cell disorder (PCD) were compared to patients with no prior PCD diagnosis. Patients with monoclonal gammopathy of undetermined signfiance (MGUS) and smoldering multiple myeloma (SMM) in the former group had lower difference between involved and uninvolved FLCs (dFLC), higher M-protein, and lower rates of t(11;14) at AL diagnosis. Patients developing AL from SMM had a shorter time to AL (median 34.2 versus 61.3 months) and higher dFLC (median 28.9 versus 11.0 mg/dl) compared to those from MGUS. Patients developing AL after known multiple myeloma (MM) or lymphoplasmacytic lymphoma (LPL) commonly lacked deep hematologic response before AL (≤ very good partial response in 78% of MM, 100% of LPL patients).

We additionally studied longitudinally followed cohorts of 3,966 MGUS and 426 (SMM) patients with longitudinal FLC measurements and matched follow-up, in which 1.8% of MGUS and 7.2% of SMM patients developed AL. Those patients who developed AL showed markedly higher dFLC at MGUS/SMM diagnosis and more frequent λ restriction and rates of t(11;14). Higher dFLC was associated with progressively earlier AL development; a 10% cumulative risk occurred at 20 months for patients with a dFLC >80 mg/dL but was not reached if dFLC <10 mg/dL at an estimated median follow-up of 86 months. In multivariable analysis, dFLC >6.4 mg/dL (HR 11.3) and λ isotype (HR 3.6) independently predicted AL, whereas heavy chain secretion was associated with lower risk (HR 0.2 for IgG).

These findings indicate that AL risk is primarily driven by cumulative light chain exposure, refining our knowledge of AL pathophysiology and providing guidance for follow-up of patients with elevated dFLC.

## 1. Introduction

Systemic light chain amyloidosis (AL) is a severe disease caused by organ deposition of misfolded free light chains (FLC) from a clonal disorder of immunoglobulin producing cells. Progressive aggregation leads to disruption of organ function and ultimately organ failure.

In population studies, MGUS preceded AL in most cases. The lifetime risk of an MGUS progressing to AL or another plasma cell malignancy is about 1-1.5% per year (1), and a skewed FLC ratio is a risk factor for progression to clinically relevant monoclonal gammopathies including AL (2). Other key precursor conditions include smoldering multiple myeloma (SMM), multiple myeloma (MM), and lymphoplasmacytic lymphoma (LPL). However, no specific factors uniquely predictive for development of AL have been identified. In practice, any significant elevation of a monoclonal FLC in a patient with a plasma cell disorder raises concern for potential light chain amyloid deposition (3-5).

Accordingly, approximately 10-15% of MM patients have concomitant AL at diagnosis (6); vice versa, about 10% of AL patients meet criteria for MM at the time AL is diagnosed (7, 8). IgM- and LPL associated AL is less common, comprising about 5-10% of AL cases overall. Patients with longstanding or relapsed MM and LPL can develop AL as a late complication (9, 10). These patients have far worse prognosis than those without AL (8, 11-15).

The plasma cell clones underlying AL show characteristic differences. In MM and LPL without AL, λ light chain isotypes are less common than κ isotypes with a 1:2 ratio, but in AL, λ clones predominate, making up approximately 75% of cases (16). Another factor prompting clinical suspicion is evidence of a t(11;14). It is common in AL clones, present in 40-50% of AL cases (17, 18), but only in 15-20% of typical myelomas and precursors (19-21).

Prior studies have focused on cross-sectional assessments at the time of AL diagnosis, since monoclonal states are usually only diagnosed during AL workup. As a result, the relative contributions of FLC elevation, duration of exposure, and clonal characteristics to the development of clinically manifest AL remain poorly defined.

To address the gap of evidence of what drives symptomatic AL development, we investigated the patterns of AL development following known monoclonal gammopathies in a large single-center cohort.

## 2. Methods

### Ethics approval and consent to participate

This retrospective study was deemed exempt by the Mayo Clinic Institutional Review Board (ID 25-004277). Patients who specifically requested that their clinical notes not be used for research were excluded. All proceedings are in accordance with the Declaration of Helsinki and other relevant guidelines and regulations.

### Study cohort

**Figure 1:**
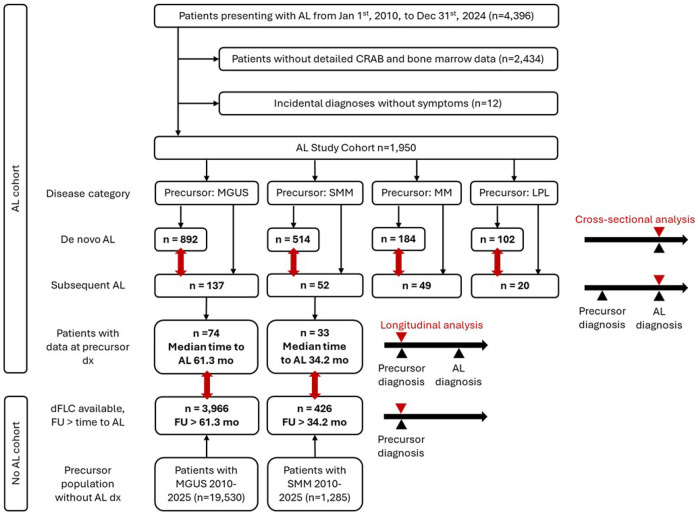
Consort diagram of patient cohorts. Flow diagram starting at the top depicts patient selection from all individuals presenting with AL between 2010 and 2025, exclusions due to missing diagnostic data or incidental, asymptomatic findings, and the resulting cross sectinoal analytic cohort. Patients are stratified by underlying clonal precursor disorder by marrow phenotype and categorized according to whether or now patient at time of AL diagnosis had a known precursor diagnosis (subsequent AL and *de novo* AL, respectively). Flow diagram starting at the bottom depicts patient selection in the cohort followed longitudinally for their precursor diagnosis. Red arrows indicate comparator groups and timepoint of analyses.

We retrospectively analyzed all AL cases diagnosed between January 1, 2010, and December 31, 2024. During this period, 4396 AL patients were evaluated at our center and provided research authorization. Systemic AL was defined by proof of a corresponding monoclonal plasma cell or lymphoplasmacytic disorder in their bone marrow and blood. Patients without proof of systemic disease were considered localized AL cases and excluded from the analysis. A total of 2446 patients were excluded due to missing detailed CRAB or bone marrow data (n=2434), or incidental asymptomatic diagnoses (n=12), yielding a final study cohort of 1950 patients (figure 1). Patients with *de novo* systemic AL amyloidosis were parsed by clonal burden: MGUS-marrow, SMM-marrow, concurrent MM by IMWG criteria (22), and concurrent LPL. We furthermore analyzed 19530 patients that were longitudinally followed with MGUS and 1285 patients longitudinally followed with SMM after January 1, 2010. We excluded patients that had a follow-up less than the median time to AL development in our respective cohorts, to ensure comparability. This approach resulted in 3966 patients followed up with MGUS and 426 patients with SMM.

### Data assessment

Data extraction from the electronic medical record was performed. Baseline variables captured included age, sex, precursor disease subtype, time from precursor diagnosis to AL, bone marrow plasma cell percentage, light chain isotype, dFLC, M-protein concentration, organ involvement, onset of AL-related symptoms, prior therapies, responses, response state at AL diagnosis, and cytogenetic abnormalities. These were assessed at the time of AL diagnosis in the cross-sectional cohort and at the time of MGUS/SMM diagnosis in the longitudinally followed cohort. Outcome measures included organ involvement at AL diagnosis, cardiac stage, overall survival (OS), and follow-up duration.

AL diagnoses in the cross-sectional cohort were categorized in respect to their plasma cell disorder as “*de novo*” or “subsequent”. An AL diagnosis was classified as subsequent if (1) it was established more than 3 months after the monoclonal disorder, and (2) there were no AL-specific symptoms or suggestive markers (elevated troponin/NT-proBNP, albuminuria) at the time of initial monoclonal gammopathy diagnosis. It was categorized as *de novo* AL if (1) AL and underlying plasma cell disorder were diagnosed within three months of each other or (2) AL was revealed during first-line therapy due to complications or (3) the AL diagnosis was suspected initially but only established by biopsy later. Cases with incidental AL findings in asymptomatic patients were excluded. MGUS and SMM include IgM cases unless an LPL phenotype was genetically confirmed as proposed (15). LPL included treated, and untreated (asymptomatic and watch-and-wait) cases. Patients with underlying marginal zone lymphoma, mantle cell lymphoma, or MALT lymphoma were excluded due to low numbers. Cases with missing data on AL or MM diagnosis dates and missing data of bone marrow plasma cells and CRAB criteria were excluded. Organ involvement was assessed as proposed by ISA (23, 24).

### Statistical analysis

Relationships between clinical, laboratory, and cytogenetic variables and timing of AL diagnosis were assessed. Differences between groups were analyzed using Fisher’s exact test for nominal variables and Kruskal Wallis for continuous, not normally distributed variables. Survival and follow-up times were estimated using Kaplan-Meier curves; differences were estimated using Log-Rank. Univariate and multivariate analyses were done using Cox proportional hazards modeling. Variables were selected based on clinical relevance and univariable significance. Analyses were conducted using complete cases; no imputation was performed. Receiver operating characteristic (ROC) analyses were performed to evaluate the discriminatory ability of baseline difference between involved and uninvolved FLCs (dFLC) for subsequent AL development, and optimal thresholds were identified using the Youden index. No formal adjustment for multiple comparisons was performed, as analyses were considered exploratory. *P* values less than 0.05 were considered significant. Patients were followed until diagnosis of AL or last clinical follow-up. For time to AL diagnosis analyses, both death and last follow up were censoring events. JMP18 (SAS) was used for statistical analysis.

## 3. Results

### Study population and baseline characteristics of the cross-sectional cohort

Of the 1,950 patients with systemic AL amyloidosis diagnosed between January 1, 2010, and December 31, 2024, 258 (13.2%) had an identified pre-existing plasma cell disorder. Of these, 137 fulfilled criteria for MGUS (53.1%), 52 for SMM (20.2%), 49 for MM (19.0%), and 20 (7.7%) for LPL (figure 1).

Of the 1029 AL patients with underlying MGUS-marrow phenotype, 892 (86.7%) had *de novo* presentations and 137 (13.3%) had prior MGUS diagnosis. For patients with prior MGUS subsequently developing AL, the median time between the diagnoses was 61.3 months (table 1). Comparing characteristics at the time of AL diagnosis of patients with subsequent AL to the *de novo* cases of AL with MGUS-marrow phenotype at evaluation, the subsequent AL group was significantly older (median 70 versus 64 years), had higher bone marrow plasma cell burden (median 9 versus 6%), lower dFLC (11.0 versus 16.1 mg/dL), higher M-protein levels (0.62 versus 0.0 g/dL) and lower rates of t(11;14) (41.7 versus 59.8%), though FISH data were available for only 53.2 and 43.8% of patients the respective groups. The subsequent group was also less likely to have hepatic involvement (5.8% versus 12.6%).

Among the 566 AL patients with underlying SMM-marrow phenotype, 514 (90.8%) had *de novo* presentations and 52 (9.2%) had prior SMM diagnosis. The major differences of baseline characteristics between *de novo* AL with SMM-marrow phenotype and subsequent AL groups were that the subsequent group had lower bone marrow plasma cell burden (median 15 versus 20%), lower dFLC (28.9 versus 41.3 mg/dL), a higher level of intact immunoglobulin (M-protein 1.4 versus 0.3 g/dL) and was less likely to have kidney involvement (27% versus 39%).

The *de novo* AL patients with SMM-marrow phenotype, as compared to the MGUS-marrow phenotype, had higher dFLC levels (41.3 versus 16.0 mg/dL) and a relatively shorter time from clonal diagnosis to AL (median 34.2 months versus 61.3 months, table 1 and figure S1a).

Of the 1950 patients with systemic AL amyloidosis, 233 (11.9%) had co-existent MM. Of these, 49 (21%) evolved from a prior active MM diagnosis. The major differences at the time of AL diagnosis between the *de novo* AL where MM was revealed at workup and the AL that subsequently developed from a prior MM diagnosis were that the subsequent cases were older (median 68 versus 65 years), had lower dFLC (median 23.9 versus 69.0 mg/dL) and BMPC (10% versus 40%) due to MM treatment, and were more likely to have cardiac involvement (57.1% versus 41.3%), but lower overall organ involvement. The median time from MM diagnosis to AL was 50.0 months figure S1a).

Six percent (122/1,950) of the systemic AL cohort had LPL-marrow phenotypes, and 20 (22%) developed AL subsequently after LPL diagnosis. Of those, 70% (14/20) had received treatment, and 30% (6/20) developed AL from asymptomatic LPL during watch-and-wait. Median time from clonal diagnosis was 42.2 months for patients that had not received treatment and 42.4 months for those after treatment. There were no significant baseline differences between *de novo* diagnosed patients and those with late AL development except for a higher rate of attributed neurologic involvement in the subsequent group (50% versus 15%, table 2).

### Risk factors for developing AL from pre-existing plasma cell disorders in the longitudinal cohort

To identify predictors of earlier AL development, we also assessed clinical characteristics at precursor diagnosis of 3966 MGUS and 426 SMM longitudinally followed patients with available FLC, no subsequent AL diagnosis and a follow-up of at least 61.3 months for patients with MGUS, and 34.2 months for SMM (figure 1), pooling them with the subsequent AL patients who had available data at the time of their precursor condition (74 MGUS-marrow AL and the 33 SMM-marrow AL). Among MGUS patients, those who developed AL were more likely to have λ light chain restriction (74.3% versus 26.1%), had markedly elevated baseline dFLC levels (median 21.75 versus 1.4 mg/dL), and were more likely to carry a t(11;14) (48.0 versus 24.9%, table 3). Patients with SMM showed the same pattern. Those who developed AL were also more likely to have lambda light chain restriction (81.8 versus 30.3%), had higher median dFLC (31.4 versus 12.9 mg/dL), and had more often proof of t(11;14) (50 versus 25.1%, table 3). In this cohort, the median time to AL diagnosis was 21 months for patients with a prior SMM diagnosis, and 28 months for those with a prior MGUS diagnosis (figure S1b).

**Figure 2:**
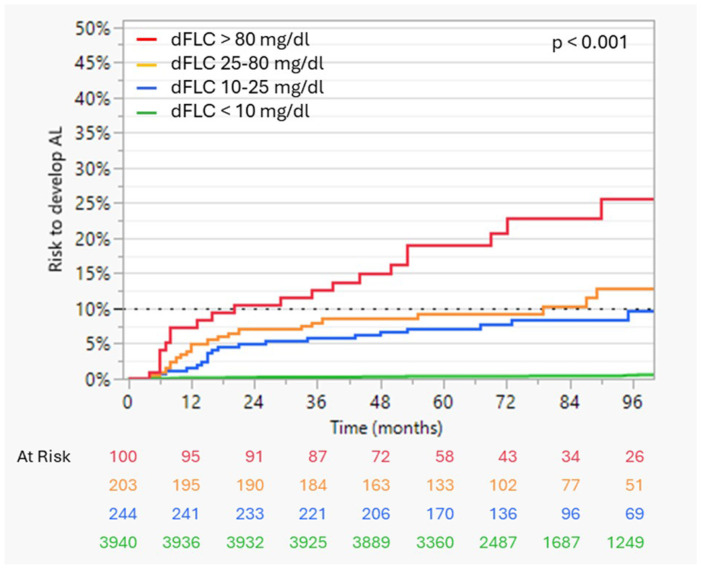
Risk of AL development for patients with MGUS and SMM by baseline dFLC levels in longitudinal cohort. Kaplan-Meier curves depicting the probability of AL diagnosis over time according to the baseline dFLC, categorized by quartiles of patients who developed AL.

**Figure 3:**
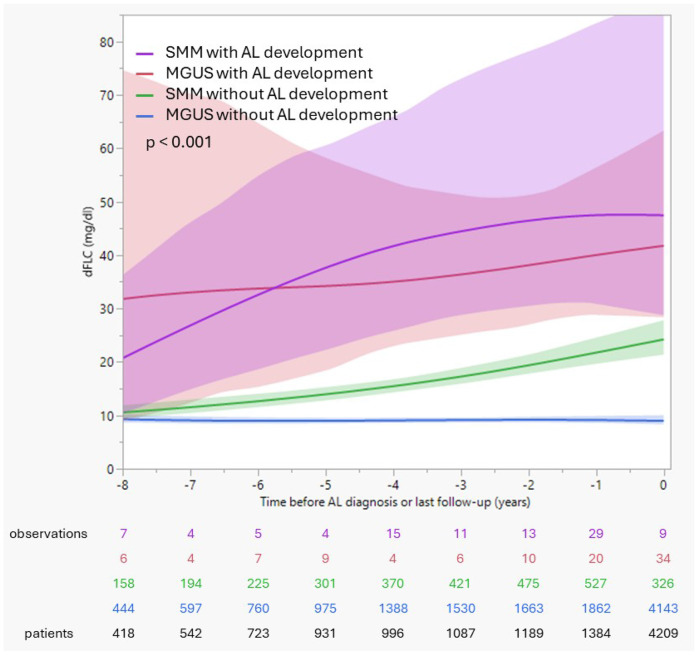
Longitudinal trajectories of serum free light chain burden prior to AL diagnosis. Smoothed trajectories of the difference between involved and uninvolved serum free light chains (dFLC, mg/dL) over time (years before AL diagnosis or last follow-up if no AL was diagnosed) are shown for patients with MGUS or SMM, stratified by subsequent development of AL. Serial FLC measurements obtained during routine follow-up of MGUS and SMM patients were analyzed. dFLC was calculated only at time points with paired κ and λ measurements. To reduce clustering of repeated tests, analyses were limited to one dFLC observation per patient per three-month window. Observation counts were aggregated by year to reflect longitudinal data completeness, and the number displayed at the 8-year timepoint represents the total observations available up to 8 years before AL diagnosis or last follow-up. Time is displayed relative to AL diagnosis (time 0) for patients who developed AL, or last follow-up or development of MM for those who did not. Solid lines represent locally smoothed mean dFLC values, with shaded areas indicating 95% confidence intervals. Numbers below the x-axis denote the number of observations contributing to each time interval.

Kaplan-Meier analysis stratified by baseline dFLC levels showed an increasing risk with higher light chain burden (figure 2). The time to a 10% cumulative risk of AL was shortest in patients with dFLC >80 mg/dL (20 months), followed by dFLC 25-80 mg/dL (79 months) and 10-25 mg/dL (95 months), and was not reached in patients with dFLC <10 mg/dL. ROC analyses identified low discriminatory dFLC thresholds of 6.4 mg/dL, associated with a 36.8-fold higher risk (95% CI 17.5–77.2) for AL development within 5 years (AUC 0.90). Parsed by precursor state, patients who developed AL amyloidosis exhibited substantially higher overall dFLC exposure than those who did not. In contrast, MGUS and SMM patients who did not develop AL maintained consistently low dFLC levels throughout follow-up (figure 3). The importance of light chain burden was also confirmed in univariable time-to-event analyses for progression to AL, where patients with elevated dFLCs, λ light chain isotype, BMPC >5%, t(11;14), IgG/IgA/IgD heavy chain secretion, and higher M-Spike had a significantly higher hazard for earlier development of AL, whereas IgM secretion was associated with lower AL risk. When these variables, excluding BMPC and t(11;14) because of substantial missingness, were entered into multivariable models, dFLC >6.4 mg/dL and λ isotype remained independently associated with an increased risk of AL development, whereas the presence of heavy chain secretion was independently associated with a reduced risk (table 4). In patients with AL, higher BMPC, and lack of heavy chain secretion, but not age, gender, light chain type, higher M-Spike or proof of t(11;14) were independently associated with a dFLC elevation >6.4 mg/dL at diagnosis (table S1).

Among MM and LPL patients, suboptimal disease control before AL onset was common. In AL developing from prior diagnosed LPL, all patients had achieved less than VGPR or had not been treated for their asymptomatic LPL. Similarly, among AL patients developing from known MM, 77.6% failed to achieve VGPR prior to AL diagnosis (38/49), and 54.2% had progressive disease at AL diagnosis (26 out of 48 patients with available data).

### Survival

Only patients with AL developing from MM had markedly worse OS when assessed from the time of AL diagnosis (18.2 versus 53.4 months, P<0.001, figure S2). In other groups, mOS from AL diagnosis was similar with 46.7 versus 54.4 months in patients with AL and MGUS phenotype, 64.1 versus 36.1 months in AL with SMM phenotype, and with LPL (51.6 versus 51.7 months). Not unexpectedly and due to lead time bias, mOS from the time of precursor diagnosis was significantly longer in patients with delayed AL development compared to those with de novo diagnosed AL across all subtypes (figure S3).

## Discussion

In this analysis of patients with plasma cell disorders, we show that the risk of AL development increases with higher dFLC levels, prolonged exposure, and lambda light chain restriction across all monoclonal precursor states, providing insight into the natural history of AL.

Our data show that subsequent development of AL from a known precursor disease is a distinct phenotype not explained by early diagnosis bias, missed or delayed recognition of mild disease: organ involvement patterns, symptom-to-diagnosis intervals, and AL stage distributions were comparable between *de novo* and subsequent AL. Patients with subsequent AL had a mOS similar to those with de novo AL from the time of the AL diagnosis with the exception of those patients who developed AL after MM diagnosis. While it is recognized that patients with longstanding or relapsed MM can develop AL as a late complication (9), our data provides data for the entire spectrum of plasma cell disorders.

We demonstrate that AL arises earlier in patients with a higher light chain burden both when comparing baseline and longitudinal characteristics of patients with MGUS and SMM and when evaluating the *de novo* versus the subsequent AL patients. Among patients who developed AL, dFLC was significantly higher compared to those who did not. Comparing patients with a de novo diagnosis to those diagnosed with AL subsequently to a clonal diagnosis, the subsequent AL patients had lower light chain burden at the time of AL diagnosis. This suggests that longer exposure to free light chains was necessary in this group of patients, as levels were lower. Moreover, AL developing from a previously known MM or LPL diagnosis was associated with lack of deep responses or progressive disease at AL diagnosis. Thus, ongoing light chain output in MM or LPL is a risk factor for AL development. Taken together, these findings suggest that specifically (1) higher dFLC and (2) prolonged exposure cumulate into risk of AL development.

In patients longitudinally followed up for MGUS/SMM, 1.8% of patients followed for MGUS and 7.2% of patients observed with SMM developed AL. The observed incidence of AL among patients with SMM is higher than expected and may reflect enrichment in a tertiary center cohort. However, the optimized dFLC threshold of 6.4 mg/dL was associated with a 36.8-fold risk of AL development within 5 years, supporting closer surveillance for patients affected. Notably, many of these patients would not meet current high-risk SMM criteria, as the involved/uninvolved FLC ratio at a dFLC of 6.4 mg/dL is typically below 20, and therefore would not be considered for treatment or closer follow-up (25, 26). This relatively low threshold also shows that most AL clones are small and produce only moderate FLC levels, while some patients exhibit very elevated monoclonal FLCs over years without ever developing AL. Moreover, AL development was relatively rare in our longitudinally followed MGUS cohort, supporting the concept of clone-specific amyloidogenic propensity (27, 28) combined with intrinsic organ tropisms (29) as additional factors for AL development. In patients who did develop AL after an MGUS/SMM diagnosis, t(11;14) was found twice as often compared to those who did not. These numbers prospectively validate previous numbers from separate cohorts, which placed the frequencies of t(11;14) in 40-50% of AL patients (17, 18, 27) versus only 15-20% of typical MM (19, 20). Interestingly, this translocation has been associated with higher light chain production (25, 26), which would provide a link towards increased risk for this population. However, we did not find this association in our cohort, possibly due to detection bias; the BMPC and t(11;14) analyses were confounded by substantial missingness of data (79.5 and 89.2%, respectively) due to lack of bone marrow sampling and low overall plasma cell counts.

**Figure 4:**
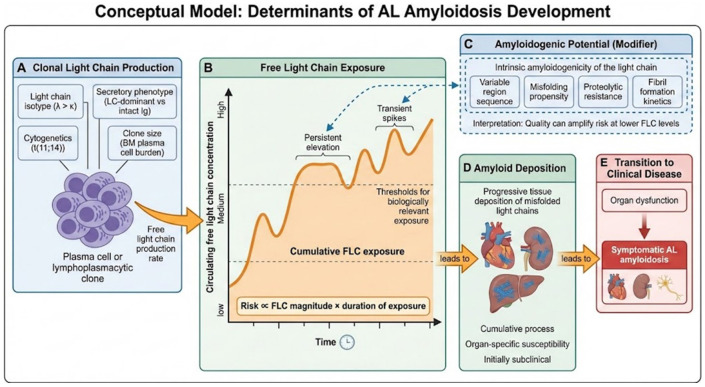
Conceptual model of risk factors for AL development (A) Clonal plasma cell or lymphoplasmacytic disorders produce monoclonal FLCs, influenced by isotype, cytogenetic features, secretory phenotype, and clone size. (B) Circulating FLC exposure varies over time and reflects both magnitude and duration of production, resulting in cumulative exposure. Persistent elevations and repeated transient spikes may exceed biologically relevant thresholds despite modest absolute FLC concentrations. (C) Intrinsic amyloidogenic properties of the light chain, determined by variable region sequence, misfolding propensity, proteolytic resistance, and fibril formation kinetics, modify the risk of amyloid formation and may amplify toxicity at lower FLC levels. (D) Sustained exposure to amyloidogenic FLCs leads to progressive tissue deposition, which is initially subclinical and organ specific. (E) Transition to symptomatic AL occurs once organ damage becomes clinically apparent.

Another risk factor for subsequent AL development in our multivariable time-to-event analyses was λ isotype. Our data support the lower amyloidogenic propensity of κ FLCs: the proportion of κ isotypes in late AL was similar despite being associated with higher dFLC levels. In vitro studies show κ FLCs may have lower inherent aggregation propensity on average (30). Clinically, κ constitutes less aggressive AL than λ (31). This suggests κ light FLCs may require more cumulative exposure for patients to develop AL. In our cohort, heavy chain secretion also was associated with lower rates of dFLC >6.4 mg/dl and a lower risk to develop AL. Intact immunoglobulin secretion may reduce the relative burden of circulating free light chains through pairing with heavy chains, and clones that invest in production of heavy chains may generate comparatively lower amounts of light chains; in addition, earlier detection and closer monitoring in patients with measurable intact M-protein could contribute to the observed association. Based on our findings, we composed a conceptual model of AL development (figure 4).

This study has several limitations. Its retrospective, single-center design limits control over data completeness. Importantly, this analysis focuses exclusively on clinically manifest, symptomatic AL and it is not suited to evaluating asymptomatic amyloid deposition. Some patients with subclinical light chain amyloid deposition may eventually progress to symptomatic disease, representing a grey zone not captured by this approach. Despite these limitations, consistent findings across multiple plasma cell types and both longitudinal and cross-sectional analyses strengthen our conclusions.

## Conclusion

Risk of AL development is driven by the cumulative burden of monoclonal FLCs, determined by both magnitude and duration of exposure. This risk is further modified by intrinsic amyloidogenic properties of the light chain, particularly λ isotype. Late AL often arises after years of exposure and is associated with the absence of deep hematologic responses in both MM and LPL. These findings refine our knowledge regarding the natural history of AL. Clinically, patients with persistent dFLC elevation, regardless of precursor disease, warrant continued vigilance for AL.

## Supplementary Material

This is a list of supplementary files associated with this preprint. Click to download.

• SupplementaryInformation.pdf

## Figures and Tables

**Table 1:** Baseline characteristics at AL diagnosis stratified by MGUS/SMM and timing of AL development in cross-sectional cohort

Variable At AL diagnosis^a^	de novo AL with MGUS phenotype (n=892)	MGUS, subsequent AL (n=137)	P	de novo AL with SMM phenotype (n=514)	SMM, subsequent AL (n=52)	P
Age at AL diagnosis (median, quartiles)	64 (58.0, 71.0)	70 (63.0, 76.5)	**<0.001**	64 (57.0, 69.0)	63 (59.0, 70.75)	0.40
Months from clonal dx to AL dx	0.20 (0, 2.2)	61.3 (17.7, 98.2)	**<0.001**	0.0 (0.0, 0.43)	34.2 (11.4, 77.2)	**<0.001**
Months from Symptom onset to AL dx	12 (6, 18.0)	13.5 (6.75, 36.0)	**0.02**	10 (5.75, 15.0)	12 (11.0, 27.5)	**0.006**
Year of diagnosis	2016 (2013, 2020)	2016 (2012, 2019)	0.10	2017 (2013, 2020)	2016 (2013, 2021)	0.30
Sex (F, %)	66.3	62.0	0.34	38.5	50.0	0.11
% Bone marrow plasma cells	6 (4.0, 10.0)	9 (5.0, 12.0)	**<0.001**	20 (15.0, 30.0)	15 (6.0, 20.0)	**<0.001**
Light chain type λ (%)	72.2 (n=809)	65.8 (n=111)	0.17	72.1 (n=463)	68.3 (n=41)	0.60
dFLC (mg/dL)	16.1 (7.1, 42.2; n=800)	11.0 (2.8, 37.6; n=125)	**0.03**	41.3 (16.6, 95.5; n=439)	28.9 (12.1, 67.0; n=41)	**0.04**
M-protein, g/dL	0.0 (0.0, 0.54; n=617)	0.62 (0.0, 1.2; n=103)	**<0.001**	0.3 (0.0, 1.1, n=359)	1.4 (0.6, 2.3, n=33)	**<0.001**
t(11;14) (%)	59.8 (n=475)	41.7 (n=60)	**0.01**	52.6 (n=308)	38.9 (n=18)	0.26
Gain/amp 1q (%)	18.1 (n=287)	26.8 (n=41)	0.20	47.9 (n=190)	29.4 (n=17)	0.14
Cardiac stage 1/2/3a/3b (%)	23/40/24/13 (n=670)	21/40/29/10 (n=90)	0.60	17/37/28/18 (n=393)	21/37/24/18 (n=33)	0.95
Palladini stage 1/2/3 (%)	53/37/10 (n=535)	55/35/10 (n=81)	0.88	61/33/6 (n=289)	59/33/8 (n=27)	0.93
Number of organs involved	1 (1, 2)	1 (0 2)	**0.008**	1 (1, 2)	1 (1, 2)	0.84
Cardiac involvement (%)	47.1	41.6	0.23	51.2	53.9	0.71
Kidney involvement (%)	49.0	40.2	0.05	42.6	21.2	**0.002**
Hepatic involvement (%)	12.6	5.8	**0.01**	10.3	5.8	0.26
GI involvement (%)	22.5	17.5	0.18	23.4	25.0	0.79
Neuro involvement (%)	16.6	13.1	0.30	13.0	21.2	0.13

a Unless otherwise stated, presented as median (interquartile range)

Table 1: Baseline characteristics at AL diagnosis stratified by precursor status and timing of AL development.

Baseline demographic, clinical, laboratory, cytogenetic, and organ involvement characteristics at the time of AL diagnosis are shown for patients with a phenotype corresponding to either monoclonal gammopathy of clinical significance (MGUS) or smoldering multiple myeloma (SMM) presenting with concurrent AL and for patients who developed AL after a prior diagnosis of MGUS or SMM. P values reflect comparisons within each precursor category between concurrent AL and AL developing after a known precursor condition. Statistically significant P values are shown in **bold.**

**Table 2:** Baseline characteristics at AL diagnosis stratified by LPL/MM and timing of AL diagnosis

Variable At AL diagnosis^A^	novo AL with MM diagnosis (n=184)	MM, subsequent AL (n=49)	P	de novo AL with LPL diagnosis (n= 102)	LPL, subsequent AL (n=20)	P
Age at AL diagnosis	65 (58.0, 71.0)	68 (58.0, 73.5)	0.17	68 (62.75, 71.25)	66 (63.0, 69.0)	0.41
Months from clonal dx to AL dx	0.16 (0.0, 2.3)	50.0 (10.0, 82.3)	**<0.001**	0 (0.0, 0.0)	42.4 (12.2, 72.1)	**<0.001**
Months from Symptom onset to AL dx	8.5 (4.0, 18.25)	10 (3.75, 18.25)	0.69	12 (6.0, 13.75)	13 (6.5, 17.75)	0.51
Year of diagnosis	2017 (2013, 2020)	2016 (2013, 2021)	0.66	2017 (2013, 2021)	2017 (2013, 2021)	0.85
Sex (F, %)	40.2	46.9	0.40	35.3	35.0	0.98
% Bone marrow plasma/lymphoplasmacytic cells	40.0 (20.0, 70.0; n=156)	10.0 (4.0, 40.0; n=35)	**<0.001**	10 (4.5, 20.0; n=89)	4.5 (2, 13.75; n=10)	0.12
Light chain type λ (%)	66.0 (n=156)	65.9 (n=44)	0.99	57.8 (n=83)	58.8 (n=17)	0.94
dFLC (mg/dL)	69.0 (16.7, 204.0; n=147)	23.9 (1.4, 147.2; n=40)	**0.001**	17.3 (5.8, 41.2; n=79)	20.6 (5.0, 56.5; n=15)	0.75
M-protein (g/dL)	0.28 (0.0, 2.0; n=168)	0.0 (0.0, 1.4; n=41)	0.15	1.1 (0.6, 1.8; n=90)	0.9 (0.3, 1.6; n=18)	0.44
t(11;14) (%)	48.4 (n=95)	46.4 (n=22)	0.30	-	-	-
Gain/amp 1q (%)	46.2 (n=65)	53.3 (n=15)	0.6	-	-	-
Cardiac stage 1/2/3a/3b (%)	20/40/28/12 (n=116)	10/55/11/24 (n=29)	**0.05**	21/47/27/5 (n=66)	25/58/17/0 (n=12)	0.61
Palladini stage 1/2/3 (%)	59/36/5 (n=111)	53/47/0 (n=32)	0.18	60/33/7 (n=52)	54/31/15 (n=13)	0.72
Number of organs involved	1 (1, 2)	1 (0, 2)	**0.01**	1 (1, 2)	1 (0, 2)	**0.08**
Cardiac involvement (%)	41.3	57.1	**0.05**	36.3	35.0	0.91
Kidney involvement (%)	32.1	40.8	0.26	36.3	50.0	0.25
Hepatic involvement (%)	6.0	6.1	0.97	3.8	15.0	0.34
GI involvement (%)	17.9	16.3	0.79	13.8	25.0	0.23
Neuro involvement (%)	14.7	14.3	0.95	14.7	50.0	**0.001**

a Unless otherwise stated, presented as median (interquartile range)

Table 2: Baseline characteristics at AL diagnosis stratified by LPL/MM and timing of AL diagnosis.

Baseline demographic, clinical, laboratory, cytogenetic, and organ involvement characteristics at the time of AL amyloidosis diagnosis are shown for patients with LPL or MM presenting with concurrent AL (LPL & AL, MM & AL) and for patients who developed AL after a prior diagnosis of LPL or MM (LPL à AL, MM à AL). P values reflect comparisons within each precursor category between concurrent AL and AL developing after a known precursor condition. Statistically significant P values are shown in **bold.**

**Table 3:** Baseline characteristics at first plasma cell disorder diagnosis: MGUS and SMM with versus without subsequent AL in the longitudinal cohort

At precursor dx^a^	MGUS, w/o AL (n=3966)	MGUS, developed AL (n=74)	P	SMM w/o AL (n=426)	SMM, developed AL (n=33)	P
Age	68.0 (60.0, 75.0)	65.0 (61.0, 73.0)	0.44	65.0 (57.0, 72.0)	60.0 (56.0, 68.0, n=33)	0.16
Sex (F, %)	39.4	33.8	0.33	41.6	42.4	0.92
% bone marrow plasma cells	6.0 (2.0, 7.0; n=498)	7.0 (4.5, 9.0; n=16)	**0.02**	20.0 (12.0, 24.7; n=385)	20.0 (10.5, 30.0; n=24)	0.40
Light chain type λ (%)	26.1 (n=3966)	74.3 (n=70)	**<0.001**	30.1 (n=426)	81.8 (n=33)	**<0.001**
dFLC (mg/dL)	1.4 (0.6, 3.0; n=3966)	21.75 (7.6, 64.0; n=64)	**<0.001**	12.9 (3.4, 40.0; n=426)	31.4 (14.5, 102.0; n=31)	**<0.001**
M-protein (g/dL)	0.0 (0.0, 0.9; n=3685)	0.0 (0.0, 0.5; n=28)	**0.02**	1.7 (0.9, 2.3; n=402)	1.7 (1.5, 2.3; n=10)	0.57
t(11;14) (%)	24.9 (n=197)	48.0 (n=25)	**0.02**	25.1 (n=251)	50.0 (n=14)	**0.05**
Gain/amp 1q (%)	13.5 (n=104)	50.0 (n=8)	0.09	32.7 (n=159)	75.0 (n=12)	**0.004**
Estimated median follow up (months, 95% CI)	85.1 (83.6-86.5)	92.8 (88.7-140.5)	**0.008**	89.9 (81.2-97.6)	96.6 (63.9-114.1)	0.76

a Unless otherwise stated, presented as median (interquartile range)

Table 3: Baseline characteristics at precursor diagnosis in patients with MGUS or SMM with and without subsequent AL in the longitudinal cohort.

Clinical, laboratory, and cytogenetic features at the time of MGUS or SMM diagnosis are shown for patients who did not develop AL during follow up versus those who developed AL. P values compare patients who developed AL with those who did not within each precursor group. Follow up time refers to observation from precursor diagnosis. Statistically significant P values are shown in **bold.**

**Table 4:** Predictors of AL development among patients with MGUS or SMM in the longitudinal cohort

Variable at MGUS/SMM diagnosis	Univariable				Multivariable^a^		
	n/N	HR	95%CI	P- value	HR	95%CI	P- value
**Higher age**	4499	0.99	0.98-1.01	0.52			
**Female gender**	1777/4499	0.87	0.58-1.30	0.50			
**BMPC >5%** ^b^	547/923	2.59	1.23-5.45	**0.007**			
**Proof of t(11;14)** ^b^	137/497	3.04	1.73-5.33	**<0.001**			
**M-protein >1.5 g/dL**	447/4154	3.23	1.90-5.50	**<0.001**	1.23	0.64-2.37	0.54
**Heavy Chain type**	4000						
No heavy chain secretion	1233	Ref	Ref	Ref	Ref	Ref	Ref
IgG	1855	1.70	1.05-2.74	**0.03**	0.19	0.09-0.38	**<0.001 0.001**
IgA	395	2.10	1.11-3.98	**0.02**	0.26	0.11-0.59	**<0.001**
IgM	513	0.29	0.088-0.97	**0.04**	0.07	0.02-0.32	0.64
IgD	4	44.19	10.4-187.8	**<0.001**	1.42	0.33-6.25	
**Light chain type λ (%)**	1245/4392	5.50	3.49-8.67	**<0.001**	3.61	2.02-6.43	**<0.001**
**dFLC >6.4 mg/dL**	806/4473	31.13	16.48-58.80	**<0.001**	11.30	5.38-23.73	**<0.001**

a 79 events

b excluded from multivariable analysis due to high missingness in event cohort

Table 4: Predictors of AL development among patients with MGUS or SMM in the longitudinal cohort.

Multivariable analysis comparing patients with MGUS or SMM who subsequently developed AL versus those who did not. N/N represents the fulfilled variable out of all available datapoints, or only available datapoints given for variables with ranges. Statistically significant P values are shown in **bold**.

## Data Availability

All data will be provided by the authors upon reasonable request.
